# A Novel Design of 4-Class BCI Using Two Binary Classifiers and Parallel Mental Tasks

**DOI:** 10.1155/2008/437306

**Published:** 2008-06-22

**Authors:** Tao Geng, John Q. Gan, Matthew Dyson, Chun SL Tsui, Francisco Sepulveda

**Affiliations:** BCI Group, Department of Computing and Electronic Systems, University of Essex, Colchester, CO4 3SQ, UK

## Abstract

A novel 4-class single-trial brain computer interface (BCI) based on two (rather than four or more) binary linear discriminant analysis (LDA) classifiers is proposed, which is called a “parallel BCI.” Unlike other BCIs where mental tasks are executed and classified in a serial way one after another, the parallel BCI uses properly designed parallel mental tasks that are executed on both sides of the subject body simultaneously, which is the main novelty of the BCI paradigm used in our experiments. Each of the two binary classifiers only classifies the mental tasks executed on one side of the subject body, and the results of the two binary classifiers are combined to give the result of the 4-class BCI. Data was recorded in experiments with both real movement and motor imagery in 3 able-bodied subjects. Artifacts were not detected or removed. Offline analysis has shown that, in some subjects, the parallel BCI can generate a higher accuracy than a conventional 4-class BCI, although both of them have used the same feature selection and classification algorithms.

## 1. Introduction

Low communication speed is one of the main problems hampering the application of brain computer interfaces (BCIs) outside laboratories. Most of the current electroencephalogram(EEG-) based BCI systems use various mental tasks which are classified and translated into different computer commands using various pattern classification algorithms. An increased number of mental tasks or brain patterns, if classified reliably, can potentially boost the communication speed of the BCI systems. This is because as the number of classes grows, the potential number of class combinations grows exponentially. In recent years, there have appeared some BCIs employing multiclass classifiers in their EEG pattern discrimination. Obermaier et al. [1] used four motor-imagery and one mental-calculation tasks. Their initial results showed that using three classes could improve the information transfer rate. With motor-imagery tasks consisting of four different classes, Naeem obtained accuracies between 33 percent and 84 percent using independent component analysis (ICA) [2]. Townsend compared common spatial patterns (CSP) with complex band power features in a four-class BCI involving motor imagery [3]. Widely used motor imagery mental tasks in 4-class BCIs [2–4] involve the movements of left hand, right hand, feet, and tongue. The tongue-related task is problematic in EEG-based BCIs because it may produce electromyography (EMG) which is difficult to monitor and could be treated as EEG by the classifiers.

To realize its potential higher information transfer rate, a multiclass BCI must have a considerably high accuracy. Unfortunately, with the number of classes increased, the accuracy of the BCIs decreases because every additional EEG pattern to be classified brings up more difficulty to the classifier. Moreover, many classification algorithms, such as linear discriminant analysis (LDA) [1] and support vector machines (SVMs), are best suited for classifying binary problems.

Although the classifiers play an important role in the accuracy of BCI systems, neurophysiological background knowledge of EEG signals, if properly exploited in the design of mental tasks and experiment paradigms, will also help improve the accuracy of a BCI system. It is well known that each hemisphere of the brain is related to the opposite side of the body. For example, left-hand movement is represented in the right motor cortex, and right hand movement in the left motor cortex. Neighboring parts of the cortex represent neighboring parts of the body. A principle used by many BCIs in choosing mental tasks is that mental tasks should activate different parts of the brain, thus generating easily separable EEG patterns.

Having in mind the basic knowledge of neurophysiology and the fact that binary classifiers greatly outperform multiclassifiers, we propose a new approach to multiple mental/motor task classification in BCI design, which we name “Parallel BCI.” The novelty in our approach lies in that two binary classifiers, called left BCI and right BCI, run in parallel to classify the properly designed parallel mental tasks that are executed simultaneously on the left side and right side of the subject body. The mental tasks of the parallel BCI only involve hand and feet movement. The results from the left BCI and right BCI are combined leading to the classification of four mental states. It is demonstrated that, in some subjects, the parallel BCI achieves a higher accuracy than the conventional 4-class BCI for classifying four mental states.

## 2. Data Acquisition

**Figure 1:**
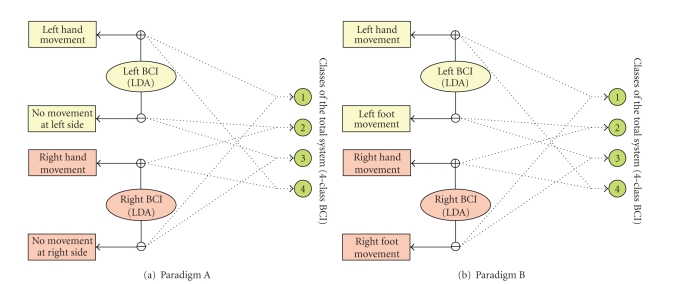
(a) The map between the two classes of the left/right BCI and the 4 classes of the whole system in paradigm A (also see Table 1(a)). The 4-class classification result of the total system is determined by the outputs of the left BCI and right BCI. For example, when and only when both left BCI and right BCI have positive outputs, the class of the total system will be regarded as 4. (b) The map in paradigm B is similar to that of paradigm A except that it involves foot movement.

**Figure 2:**
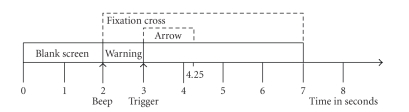
Experimental paradigm begins with a blank screen. After 2 seconds, a fixation cross appears and an audio tone warns the subject to prepare. At second three, an arrow appears on the screen, indicating the motor imagery the subject should perform (adapted from [1]).

We designed two parallel paradigms for our experiments. One only involves hands movement (paradigm A), and the other involves hand and feet movement (paradigm B). Their corresponding labels are described in Table 1 and Figure 1, respectively. The experiment consisted of 3 runs with 40 trials each for each subject. In each trial, from *t* = 3 seconds, an arrow pointing to left, right, up, or down was displayed (see Figure 2). Subjects were instructed to execute or imagine hand/foot movement at one or both sides of the body, as indicated in Table 1. For example, in the experiment of paradigm B (see Table 1(b)), when the cue of an up arrow is displayed, the subject should imagine movements of both hands at the same time. When a left arrow displayed, the subject should imagine a left-hand movement and a right-foot movement at the same time. For a right arrow, it means simultaneous right-hand movement and left-foot movement. The down arrow means simultaneous movements at both feet. Combining the movements executed simultaneously at both sides of the subject body, we can get the class labels of the 4-class whole system (see Table 1). In paradigm A, these classes (combinations) are both hands, left hand only, right hand only, no movement at all (see Table 1(a)). In paradigm B, they are both hands, left hand and right foot, right hand and left foot, both feet (see Table 1). No feedback was shown to the subject in the experiments. It should be noted that, in paradigm A, “no movement” (or relax) at left/right side of the subject body is regarded as a mental task (EEG pattern) in the left/right BCI. “Relax” has been used as an EEG pattern in synchronous BCIs, though not quite commonly. For example, Akrami et al. [5] employed baseline as a mental task in a 3-class BCI.

**Figure 3:**
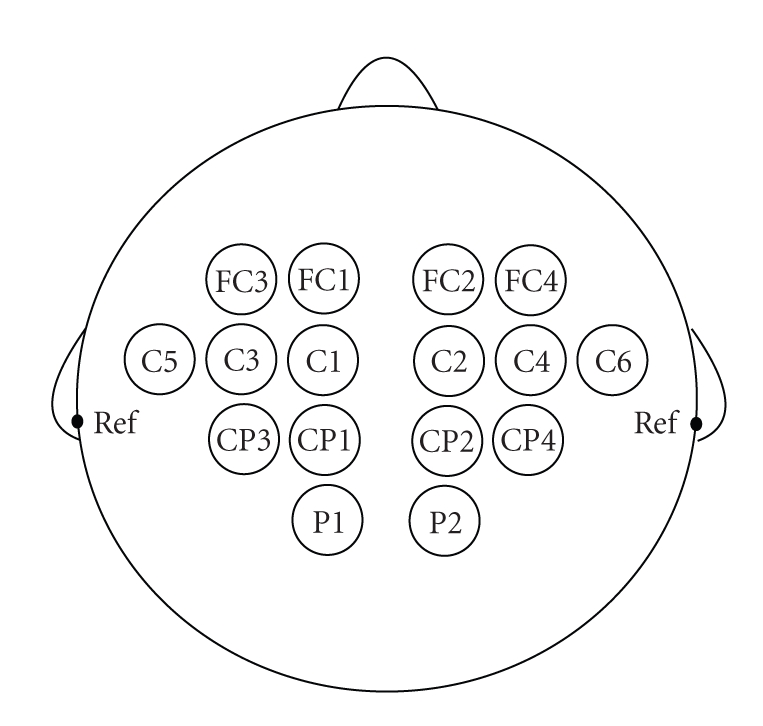
The electrode positions of the parallel BCI.

The electrode positions with respect to the international 10–20 systems are shown in Figure 3. The recording was made with a 16-channel EEG amplifier from G-Technology (http://www.gtec.at/). The channels in the left hemisphere were referenced to the left mastoid. The channels in the right hemisphere were referenced to the right mastoid. The EEG was sampled at 256 Hz.

## 3. Data Processing

The recorded EEG data was first filtered for 0.5–100 Hz, and then preprocessed with common average reference and band power feature extraction (with 16 bands covering 8–45 Hz, that is, 8-9 Hz, 10-11 Hz, 12-13 Hz, 14-15 Hz, 16-17 Hz, 18-19 Hz, 20-21 Hz, 22-23 Hz, 24-25 Hz, 26-27 Hz, 28–30 Hz, 31–33 Hz, 34–36 Hz, 37–39 Hz, 40–42 Hz, 43–45 Hz). The band power of each frequency band at each channel is calculated by first digitally bandpass filtering the data, squaring each sample and taking logarithm, and then averaging over a one-second sliding window [6]. Averaging the samples of band power over a one-second window is a method widely used in EEG-based BCIs to smooth the data and reduce the variability. Electrooculogram (EOG) and other artifacts were not detected or removed. A subset of no more than 20 features was selected using a sequential forward floating selection (SFFS) [7] algorithm based on 3-fold cross-validation. SFFS starts from an empty set and in each iteration generates new subsets by adding a feature selected by an evaluation measure (here, it is the LDA classifier) [7]. It has been found that simple linear classifiers were just marginally worse than complex nonlinear methods [8, 9]. It was shown in the BCI Competition 2003 and 2005 that LDA performed as well as (sometimes even outperforms) SVMs [10], and almost all the winning classifiers were linear [11]. Hence, two binary LDA classifiers, one in left BCI and the other in right BCI (see Figure 1), were used to classify the two motor tasks of the left side and right side, respectively. The binary LDA classifier assigns linear weights to the band power features so as to provide a separating hyperplane between the two classes in feature space. For details of the LDA algorithm, please refer to [1]. The 4-class result of the Parallel BCI was obtained according to the class label coding indicated in Table 1 and Figure 1.

**Figure 4:**
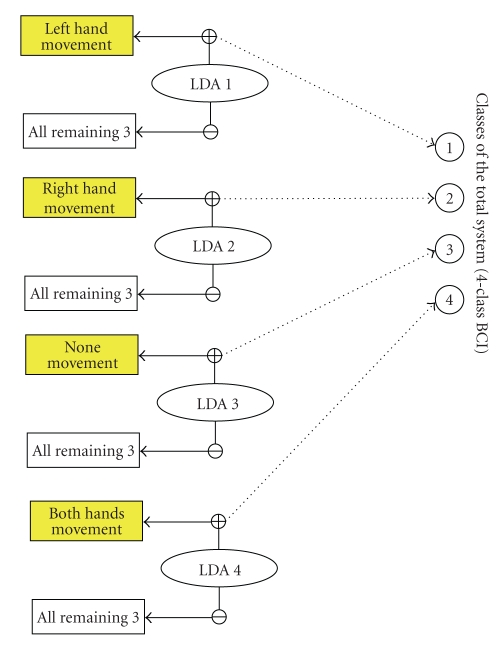
The map between the positive output of the 4 one-versus-all LDA classifier and the 4 classes of the conventional BCI (also see Table 1) in paradigm A. For example, LDA 3 is a binary classifier discriminating class 3 against all remaining classes (1, 2, 4). If its positive output is the largest among all the four binary classifiers, the class of the conventional BCI will be regarded as 3.

For a comparison, we also processed the data by regarding the system as a conventional four-class BCI, which, for convenience, is called “conventional BCI.” The 4 classes of the conventional BCI in paradigm A are the four combinations of the movements executed simultaneously on the left and right sides of the subject body (i.e., both hands, left hand only, right hand only, none movements at all) (see Figure 4). Similarly, the 4 classes of the conventional BCI in paradigm B are both hands, left hand and right foot, right hand and left foot, both feet. The conventional BCI used the same feature extraction and classification (LDA) methods. As LDA is not directly appropriate for 4-class classification, we used four one-versus-all binary LDA classifiers. Rifkin's analysis and review [12] has shown that, for multiclass problems, the “one-versus-all” scheme can be as accurate as any other approaches. In the conventional BCI, each LDA classifier was trained to discriminate one of the four classes from the remaining three. For each test sample, the four classifiers were run each with the data. The classifier that generated the largest positive value was chosen to give the result of the conventional 4-class BCI [12].

## 4. Results

Each data set was obtained from an experiment (paradigm A or B) of one subject, consisting of 3 sessions, each with 40 trials. It was processed using 3-fold cross validation. The averaged accuracy got from the test data of the three folds of each data set is shown in Tables 2 and 3. Subjects 1 and 2 are male and right-hand dominant. They had experience in BCI experiments. Subject 3 is female and left-hand dominant, and had no experience of BCI experiments before. All subjects were able-bodied. In some experiments of paradigm A and paradigm B, the parallel BCI produced a higher accuracy than that of the conventional BCI. Experiences and training for the parallel BCI experiments in Subjects 1 and 2 have incurred better results than in Subject 3.

## 5. Discussion

The novelty of the parallel BCI is in the design of the mental tasks (i.e., the coded parallel mental tasks). Unlike other BCIs where the mental tasks are executed and classified one after another in a serial way, the mental tasks in the parallel BCI are executed parallel at both sides of the subject body. Moreover, the binary mental tasks at each side of the subject body are separately classified by a binary classifier. The potential separability of the EEG patterns caused by the left and right limbs has been exploited to reduce a 4-class BCI to two binary BCIs. For some subjects, this reduction has brought the whole system, a 4-class BCI, an accuracy higher than that of a conventional 4-class BCI which employed 4 one-versus-all binary classifiers.

The parallel BCI and the conventional BCI involved in this paper have indeed used the same binary classification algorithm (LDA), the same features (band power), and the same feature selection algorithms (SFFS). The vital difference between them is that the parallel BCI has exploited the coding in the properly designed parallel mental tasks while the conventional BCI has not. Therefore, the improved performance of the parallel BCI for some subjects is due to the coded mental tasks rather than the classifier or the feature selection algorithm it used.

One drawback of the parallel BCI (especially in paradigm B involving hand and foot movements) is that the subjects need a few training sessions before they can get used to the simultaneous parallel mental tasks at their left and right hand/foot. Because this is the first time this kind of simultaneously executed mental tasks were used in a BCI study, the neurological difference between the topographic patterns of parallel mental task and serial mental task is not clear. Moreover, currently only simple band power features were used for the classification. Common spatial pattern (CSP) method has shown its efficacy in extracting topographic pattern of brain rhythm modulations [13]. Phase synchronization reflects the cooperative interactions between anatomically disparate neural populations [14]. These methods could be more appropriate for classifying the parallel mental tasks, which will be investigated in the future work.

Our current work considers only offline analysis of synchronous BCI experiments. An offline scenario is more suitable for comparing the schemes of the parallel BCI and conventional BCI as it is more reliable and stable [10, 15]. However, the aim of our next work is online BCI. As shown in other BCIs, with online feedback, the classification accuracy can be increased even more.

## Figures and Tables

**Table:** (a) Paradigm A

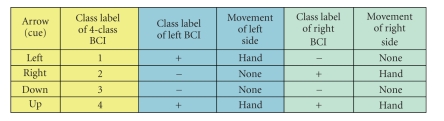

**Table:** (b) Paradigm B

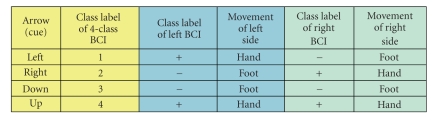

**Table 2:**
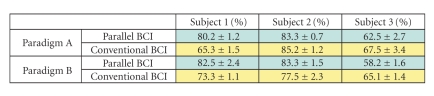
Classification accuracies (mean and standard deviation) of the parallel BCI and conventional BCI with 3 subjects executing real motor tasks (3-fold cross-validation).

**Table 3:**
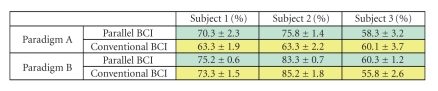
Classification accuracies (mean and standard deviation) of the parallel BCI and conventional BCI with 3 subjects executing motor imagery tasks (3-fold cross-validation).
